# Supplementary material for: The Potential of Pediococcus acidilactici Cell-Free Supernatant as a Preservative in Food Packaging Materials
Source: Foods. 2024 Feb 21;13(5):644. doi: 10.3390/foods13050644 (PMC10930656; doi:10.3390/foods13050644)
Supplement: Supplementary file 1 [file foods-13-00644-s001.zip › foods-2819111-supplementary.pdf]

Supplementary data

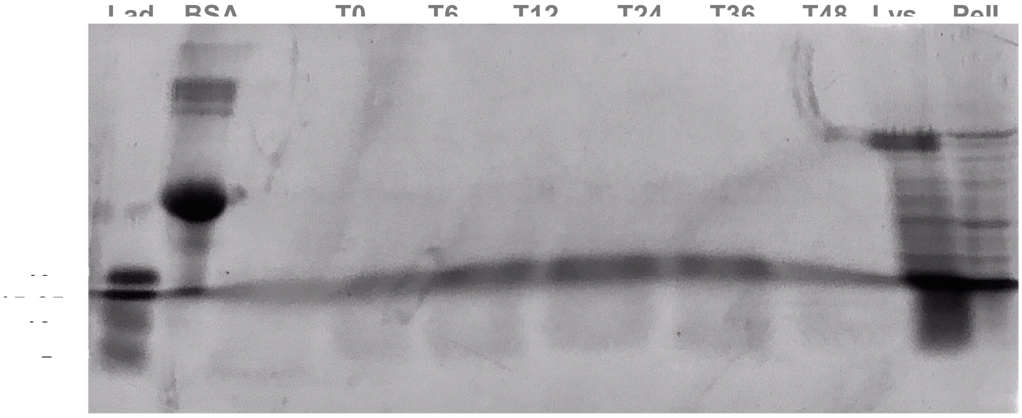

Supplementary Figure S1. SDS page visualization of *Pediococcus acidilactici* lysate, pellet, and CFS from T0-T48. Bovine Serum Albumin was used as a positive control to show that the gel could well detect any proteinaceous content in the samples loaded to the gel.

Supplementary Table S1. pH of diluted CFS stock before MIC test

CFS concentration stock (%)	pH of CFS stock	CFS concentration during MIC test (%)
100	3.75 ± 0.02	50
80	3.88 ± 0.08	40
60	4.09 ± 0.01	30
40	4.37 ± 0.02	20
20	4.44 ± 0.07	10
10	5.16 ± 0.01	5

Supplementary Table S2. Statistical analysis from Figure 2

2a. ***S. aureus***

ANOVA table	SS	DF	MS	F (DFn, DFd)	P value
Treatment (between columns)	386.3	6	64.39	F (6, 12) = 6239	P<0.0001
Residual (within columns)	0.1238	12	0.01032		
Total	386.4	18			

Tukey's multiple comparisons test	Mean Diff.	95.00% CI of diff.	Significant?	Summary	Adjusted P Value
Initial vs. CFS	5.673	5.383 to 5.964	Yes	****	<0.0001
Initial vs. CFS-N	-3.19	-3.480 to -2.900	Yes	****	<0.0001
Initial vs. CFS-P	5.673	5.383 to 5.964	Yes	****	<0.0001

Initial vs. CFS-C	5.673	5.383 to 5.964	Yes	****	<0.0001
Initial vs. MRSB	-4.442	-4.766 to -4.117	Yes	****	<0.0001
Initial vs. MHB	-5.142	-5.466 to -4.817	Yes	****	<0.0001
CFS vs. CFS-N	-8.863	-9.154 to -8.573	Yes	****	<0.0001
CFS vs. CFS-P	0	-0.2903 to 0.2903	No	ns	>0.9999
CFS vs. CFS-C	0	-0.2903 to 0.2903	No	ns	>0.9999
CFS vs. MRSB	-10.12	-10.44 to -9.790	Yes	****	<0.0001
CFS vs. MHB	-10.82	-11.14 to -10.49	Yes	****	<0.0001
CFS-N vs. CFS-P	8.863	8.573 to 9.154	Yes	****	<0.0001
CFS-N vs. CFS-C	8.863	8.573 to 9.154	Yes	****	<0.0001
CFS-N vs. MRSB	-1.252	-1.576 to -0.9271	Yes	****	<0.0001
CFS-N vs. MHB	-1.952	-2.276 to -1.627	Yes	****	<0.0001
CFS-P vs. CFS-C	0	-0.2903 to 0.2903	No	ns	>0.9999
CFS-P vs. MRSB	-10.12	-10.44 to -9.790	Yes	****	<0.0001
CFS-P vs. MHB	-10.82	-11.14 to -10.49	Yes	****	<0.0001
CFS-C vs. MRSB	-10.12	-10.44 to -9.790	Yes	****	<0.0001
CFS-C vs. MHB	-10.82	-11.14 to -10.49	Yes	****	<0.0001
MRSB vs. MHB	-0.7	-1.056 to -0.3445	Yes	***	0.0002

2b. ***EHEC***

ANOVA table	SS	DF	MS	F (DFn, DFd)	P value
Treatment (between columns)	409.3	6	68.22	F (6, 14) = 3112	P<0.0001
Residual (within columns)	0.3069	14	0.02192		
Total	409.6	20			

Tukey's multiple comparisons test	Mean Diff.	95.00% CI of diff.	Significant?	Summary	Adjusted P Value
Initial vs. CFS	6.897	6.484 to 7.309	Yes	****	<0.0001
Initial vs. CFS-N	-2.7	-3.113 to -2.287	Yes	****	<0.0001
Initial vs. CFS-P	6.897	6.484 to 7.309	Yes	****	<0.0001
Initial vs. CFS-C	6.897	6.484 to 7.309	Yes	****	<0.0001
Initial vs. MRSB	-1.77	-2.183 to -1.357	Yes	****	<0.0001
Initial vs. MHB	-2.927	-3.339 to -2.514	Yes	****	<0.0001
CFS vs. CFS-N	-9.597	-10.01 to -9.184	Yes	****	<0.0001
CFS vs. CFS-P	0	-0.4128 to 0.4128	No	ns	>0.9999
CFS vs. CFS-C	0	-0.4128 to 0.4128	No	ns	>0.9999
CFS vs. MRSB	-8.667	-9.079 to -8.254	Yes	****	<0.0001
CFS vs. MHB	-9.823	-10.24 to -9.411	Yes	****	<0.0001
CFS-N vs. CFS-P	9.597	9.184 to 10.01	Yes	****	<0.0001
CFS-N vs. CFS-C	9.597	9.184 to 10.01	Yes	****	<0.0001
CFS-N vs. MRSB	0.93	0.5172 to 1.343	Yes	****	<0.0001
CFS-N vs. MHB	-0.2267	-0.6394 to 0.1861	No	ns	0.525
CFS-P vs. CFS-C	0	-0.4128 to 0.4128	No	ns	>0.9999
CFS-P vs. MRSB	-8.667	-9.079 to -8.254	Yes	****	<0.0001
CFS-P vs. MHB	-9.823	-10.24 to -9.411	Yes	****	<0.0001
CFS-C vs. MRSB	-8.667	-9.079 to -8.254	Yes	****	<0.0001
CFS-C vs. MHB	-9.823	-10.24 to -9.411	Yes	****	<0.0001
MRSB vs. MHB	-1.157	-1.569 to -0.7439	Yes	****	<0.0001

2c. ***L. monocytogenes***

ANOVA table	SS	DF	MS	F (DFn, DFd)	P value
Treatment (between columns)	326	6	54.34	F (6, 14) = 3865	P<0.0001
Residual (within columns)	0.1968	14	0.01406		
Total	326.2	20			

Tukey's multiple comparisons test	Mean Diff.	95.00% CI of diff.	Significant?	Summary	Adjusted P Value
Initial vs. CFS	5.367	5.036 to 5.697	Yes	****	<0.0001
Initial vs. CFS-N	-3.853	-4.184 to -3.523	Yes	****	<0.0001
Initial vs. CFS-P	5.367	5.036 to 5.697	Yes	****	<0.0001
Initial vs. CFS-C	5.367	5.036 to 5.697	Yes	****	<0.0001
Initial vs. MRSB	-2.667	-2.997 to -2.336	Yes	****	<0.0001
Initial vs. MHB	-2.67	-3.001 to -2.339	Yes	****	<0.0001
CFS vs. CFS-N	-9.22	-9.551 to -8.889	Yes	****	<0.0001
CFS vs. CFS-P	0	-0.3306 to 0.3306	No	ns	>0.9999
CFS vs. CFS-C	0	-0.3306 to 0.3306	No	ns	>0.9999
CFS vs. MRSB	-8.033	-8.364 to -7.703	Yes	****	<0.0001
CFS vs. MHB	-8.037	-8.367 to -7.706	Yes	****	<0.0001
CFS-N vs. CFS-P	9.22	8.889 to 9.551	Yes	****	<0.0001
CFS-N vs. CFS-C	9.22	8.889 to 9.551	Yes	****	<0.0001
CFS-N vs. MRSB	1.187	0.8561 to 1.517	Yes	****	<0.0001
CFS-N vs. MHB	1.183	0.8528 to 1.514	Yes	****	<0.0001
CFS-P vs. CFS-C	0	-0.3306 to 0.3306	No	ns	>0.9999
CFS-P vs. MRSB	-8.033	-8.364 to -7.703	Yes	****	<0.0001
CFS-P vs. MHB	-8.037	-8.367 to -7.706	Yes	****	<0.0001

CFS-C vs. MRSB	-8.033	-8.364 to -7.703	Yes	****	<0.0001
CFS-C vs. MHB	-8.037	-8.367 to -7.706	Yes	****	<0.0001
MRSB vs. MHB	-0.003333	-0.3339 to 0.3272	No	ns	>0.9999

Supplementary Table S3. Statistical analysis from Figure 3

**3a.** ***S. aureus***					
ANOVA table	SS	DF	MS	F (DFn, DFd)	P value
Treatment (between columns)	213.9	4	53.46	F (4, 10) = 3396	P<0.0001
Residual (within columns)	0.1574	10	0.01574		
Total	214	14			
Tukey's multiple comparisons test	Mean Diff.	95.00% CI of diff.	Significant?	Summary	Adjusted P Value
Initial vs. CFS	5.91	5.573 to 6.247	Yes	****	<0.0001
Initial vs. CFS-N	-2.954	-3.291 to -2.617	Yes	****	<0.0001
Initial vs. MRSB	-1.713	-2.050 to -1.376	Yes	****	<0.0001
Initial vs. Lactic Acid	5.91	5.573 to 6.247	Yes	****	<0.0001
CFS vs. CFS-N	-8.864	-9.201 to -8.527	Yes	****	<0.0001
CFS vs. MRSB	-7.623	-7.960 to -7.286	Yes	****	<0.0001
CFS vs. Lactic Acid	0	-0.3372 to 0.3372	No	ns	>0.9999
CFS-N vs. MRSB	1.241	0.9038 to 1.578	Yes	****	<0.0001
CFS-N vs. Lactic Acid	8.864	8.527 to 9.201	Yes	****	<0.0001
MRSB vs. Lactic Acid	7.623	7.286 to 7.960	Yes	****	<0.0001

**3b.** ***EHEC***					
ANOVA table	SS	DF	MS	F (DFn, DFd)	P value
Treatment (between columns)	5155	9	572.8	F (9, 20) = 46.35	P<0.0001
Residual (within columns)	247.2	20	12.36		
Total	5403	29			
Tukey's multiple comparisons test	Mean Diff.	95.00% CI of diff.	Significant?	Summary	Adjusted P Value
Initial vs. CFS	7.878	7.639 to 8.117	Yes	****	<0.0001
Initial vs. CFS-N	-1.72	-1.959 to -1.481	Yes	****	<0.0001
Initial vs. MRSB	-3.408	-3.647 to -3.169	Yes	****	<0.0001

Initial vs. Lactic Acid	2.078	1.839 to 2.317	Yes	****	<0.0001
CFS vs. CFS-N	-9.598	-9.837 to -9.359	Yes	****	<0.0001
CFS vs. MRSB	-11.29	-11.52 to -11.05	Yes	****	<0.0001
CFS vs. Lactic Acid	-5.8	-6.039 to -5.561	Yes	****	<0.0001
CFS-N vs. MRSB	-1.688	-1.927 to -1.449	Yes	****	<0.0001
CFS-N vs. Lactic Acid	3.798	3.559 to 4.037	Yes	****	<0.0001
MRSB vs. Lactic Acid	5.486	5.247 to 5.725	Yes	****	<0.0001

3c. ***L. monocytogenes***

ANOVA table	SS	DF	MS	F (DFn, DFd)	P value
Treatment (between columns)	249.7	4	62.43	F (4, 10) = 4091	P<0.0001
Residual (within columns)	0.1526	10	0.01526		
Total	249.9	14			

Tukey's multiple comparisons test	Mean Diff.	95.00% CI of diff.	Significant?	Summary	Adjusted P Value
Initial vs. CFS	6.877	6.545 to 7.209	Yes	****	<0.0001
Initial vs. CFS-N	-2.342	-2.674 to -2.010	Yes	****	<0.0001
Initial vs. MRSB	-1.581	-1.913 to -1.249	Yes	****	<0.0001
Initial vs. Lactic Acid	6.877	6.545 to 7.209	Yes	****	<0.0001
CFS vs. CFS-N	-9.219	-9.551 to -8.887	Yes	****	<0.0001
CFS vs. MRSB	-8.458	-8.790 to -8.126	Yes	****	<0.0001
CFS vs. Lactic Acid	0	-0.3319 to 0.3319	No	ns	>0.9999
CFS-N vs. MRSB	0.761	0.4291 to 1.093	Yes	***	0.0001
CFS-N vs. Lactic Acid	9.219	8.887 to 9.551	Yes	****	<0.0001
MRSB vs. Lactic Acid	8.458	8.126 to 8.790	Yes	****	<0.0001

Supplementary Table S4. Statistical analysis from Figure 5

ANOVA table	SS	DF	MS	F (DFn, DFd)	P value
Treatment (between columns)	5155	9	572.8	F (9, 20) = 46.35	P<0.0001
Residual (within columns)	247.2	20	12.36		
Total	5403	29			

Tukey's multiple comparisons test	Mean Diff.	95.00% CI of diff.	Significant?	Summary	Adjusted P Value
CFS 100% vs. CFS 90%	-4.703	-14.87 to 5.461	No	ns	0.8148

CFS 100% vs. CFS 80%	-0.1666	-10.33 to 9.998	No	ns	>0.9999
CFS 100% vs. CFS 70%	0.3033	-9.861 to 10.47	No	ns	>0.9999
CFS 100% vs. CFS 60%	9.351	-0.8134 to 19.52	No	ns	0.0876
CFS 100% vs. CFS 50%	13.06	2.899 to 23.23	Yes	**	0.0058
CFS 100% vs. CFS 40%	15.38	5.214 to 25.54	Yes	***	0.001
CFS 100% vs. CFS 30%	32.77	22.61 to 42.94	Yes	****	<0.0001
CFS 100% vs. CFS 20%	31.07	20.91 to 41.24	Yes	****	<0.0001
CFS 100% vs. CFS 10%	26.02	15.86 to 36.19	Yes	****	<0.0001
CFS 90% vs. CFS 80%	4.537	-5.628 to 14.70	No	ns	0.8425
CFS 90% vs. CFS 70%	5.007	-5.158 to 15.17	No	ns	0.7595
CFS 90% vs. CFS 60%	14.05	3.890 to 24.22	Yes	**	0.0027
CFS 90% vs. CFS 50%	17.77	7.602 to 27.93	Yes	***	0.0002
CFS 90% vs. CFS 40%	20.08	9.918 to 30.25	Yes	****	<0.0001
CFS 90% vs. CFS 30%	37.48	27.31 to 47.64	Yes	****	<0.0001
CFS 90% vs. CFS 20%	35.78	25.61 to 45.94	Yes	****	<0.0001
CFS 90% vs. CFS 10%	30.73	20.56 to 40.89	Yes	****	<0.0001
CFS 80% vs. CFS 70%	0.4699	-9.695 to 10.63	No	ns	>0.9999
CFS 80% vs. CFS 60%	9.518	-0.6468 to 19.68	No	ns	0.0783
CFS 80% vs. CFS 50%	13.23	3.065 to 23.39	Yes	**	0.0051
CFS 80% vs. CFS 40%	15.55	5.381 to 25.71	Yes	***	0.0009
CFS 80% vs. CFS 30%	32.94	22.78 to 43.10	Yes	****	<0.0001
CFS 80% vs. CFS 20%	31.24	21.08 to 41.40	Yes	****	<0.0001
CFS 80% vs. CFS 10%	26.19	16.03 to 36.36	Yes	****	<0.0001
CFS 70% vs. CFS 60%	9.048	-1.117 to 19.21	No	ns	0.1072
CFS 70% vs. CFS 50%	12.76	2.596 to 22.92	Yes	**	0.0074
CFS 70% vs. CFS 40%	15.08	4.911 to 25.24	Yes	**	0.0013
CFS 70% vs. CFS 30%	32.47	22.31 to 42.63	Yes	****	<0.0001
CFS 70% vs. CFS 20%	30.77	20.61 to 40.93	Yes	****	<0.0001
CFS 70% vs. CFS 10%	25.72	15.56 to 35.89	Yes	****	<0.0001
CFS 60% vs. CFS 50%	3.712	-6.452 to 13.88	No	ns	0.944
CFS 60% vs. CFS 40%	6.028	-4.137 to 16.19	No	ns	0.5473
CFS 60% vs. CFS 30%	23.42	13.26 to 33.59	Yes	****	<0.0001

CFS 60% vs. CFS 20%	21.72	11.56 to 31.89	Yes	****	<0.0001
CFS 60% vs. CFS 10%	16.67	6.509 to 26.84	Yes	***	0.0004
CFS 50% vs. CFS 40%	2.315	-7.849 to 12.48	No	ns	0.9976
CFS 50% vs. CFS 30%	19.71	9.546 to 29.87	Yes	****	<0.0001
CFS 50% vs. CFS 20%	18.01	7.846 to 28.17	Yes	***	0.0001
CFS 50% vs. CFS 10%	12.96	2.796 to 23.13	Yes	**	0.0063
CFS 40% vs. CFS 30%	17.4	7.231 to 27.56	Yes	***	0.0002
CFS 40% vs. CFS 20%	15.69	5.530 to 25.86	Yes	***	0.0008
CFS 40% vs. CFS 10%	10.65	0.4810 to 20.81	Yes	*	0.0355
CFS 30% vs. CFS 20%	-1.7	-11.86 to 8.464	No	ns	0.9998
CFS 30% vs. CFS 10%	-6.75	-16.91 to 3.415	No	ns	0.401
CFS 20% vs. CFS 10%	-5.049	-15.21 to 5.115	No	ns	0.7513
